# Infection: a Cause of and Cure for Cancer

**DOI:** 10.1007/s40495-017-0109-y

**Published:** 2017-10-05

**Authors:** Jenna H. Newman, Andrew Zloza

**Affiliations:** 1 Section of Surgical Oncology Research, Division of Surgical Oncology, Rutgers Cancer Institute of New Jersey, 195 Little Albany Street, New Brunswick, NJ 08903 USA; 2 Department of Surgery, Rutgers Robert Wood Johnson Medical School, Rutgers, The State University of New Jersey, 675 Hoes Lane West, Piscataway Township, NJ 08854 USA

**Keywords:** Infection, Inflammation, Pathogen, Cancer, Oncolytic, Oncogenic, Non-oncolytic, Non-oncogenic

## Abstract

**Purpose of Review:**

This article provides a brief overview of the role that infections play in cancer emergence and cancer treatment.

**Recent Findings:**

A select number of pathogens have been reported to increase the incidence of specific cancers (directly through altering gene expression or indirectly through inducing chronic inflammation). These have been referred to as oncogenic pathogens. Conversely, a subset of pathogens has been demonstrated to preferentially cause lysis of tumor cells, leading to tumor regression and improved anti-tumor immunity. These have been termed oncolytic pathogens. However, the contribution of non-oncogenic, non-oncolytic pathogens to both tumor growth and regression is likewise being increasingly recognized.

**Summary:**

Pathogens have both the ability to cause and cure cancer. However, the mechanisms underlying these pathogen-mediated outcomes are not fully understood. With the recent emergence of interest in the immunotherapy of cancer, it is important that future studies focus specifically on preventing the negative effects of oncogenic infections, deconstructing the positive role of oncolytic pathogens, and finally providing insight into the dual roles of non-oncolytic, non-oncogenic pathogens so that anti-pathogen immune responses can be harnessed as a transformative means to treat cancer.

## Introduction

**Fig. 1:**
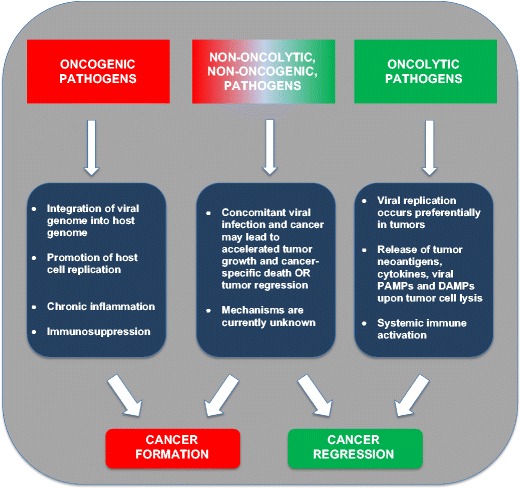
Role of infections in cancer. This schematic describes the manner in which oncogenic, oncolytic, and non-oncogenic, non-oncolytic pathogens affect cancer formation and cancer regression

Case reports dating to the ancient past and more recent epidemiological studies have described both a positive and negative role for infection in the context of cancer (Fig. 1). In ancient Egypt, a physician named Imhotep reported regression of tumors in which infection was initiated by making an incision at the tumor site [1]. Similar reports emerged throughout the first millennium, but remained isolated observations until American physician William Coley, inspired by earlier reports and his own observations, began to systematically study the relationship between infection and tumor regression in the late nineteenth century [1]. In 1891, Coley observed the complete regression of a sarcoma after failure of a surgical wound to close [1]. Coley hypothesized that this regression was enabled by the patient’s febrile erysipelas infection, caused by the bacterium *Streptococcus pyogenes* [1]. After conducting a series of experiments, Coley developed a vaccine that harbored toxins from killed *Streptococcus pyogenes* and *Serratia marcescens* bacteria [1]. With variable success across patients, Coley’s approach waned in popularity upon the advent of radiation and chemotherapy as cancer treatments [1]. However, interest in the positive role that infections can play in tumor regression has experienced resurgence in recent years with the discovery that some pathogens, specifically oncolytic viruses, can preferentially replicate and lyse cancer cells [2].

In the early twentieth century, evidence for a link between infection and cancer was made when growth of human warts and chicken leukemia could be initiated in naïve hosts subsequent to transfer of cell-free extracts from lesions of afflicted hosts [3–5]. However, due to the fact that leukemia was not considered to be a malignancy at the time of publishing, these results were overlooked. Upon realization that a component of a cell-free extract (virus particles) could trigger the development of cancer in a recipient animal, the concept of oncogenic (cancer-causing) viruses emerged. Throughout the twentieth century, dozens of oncogenic viruses, many employing distinct mechanisms to promote cancer development, were identified. As there has been growing understanding of the complex relationship between infection and cancer, it has become clear that infection with non-oncogenic, non-oncolytic pathogens can impact tumor growth. For instance, it has been reported that patients afflicted with non-gastrointestinal acute bacterial infections exhibited higher rates of colon cancer-specific death compared to those without bacterial illness [6].

Although conflicting reports regarding the role of infection in tumor development and cancer cure exist, it is widely accepted that a select group of pathogens increase the incidence of cancer (oncogenic pathogens) and that a select group of pathogens preferentially lyse tumor cells and lead to tumor regression (oncolytic pathogens). However, it should be noted that while select pathogens may curtail tumor growth, to our knowledge, no viral infection has been shown to prevent cancer in humans. In this review, we focus on the role of these two subsets of pathogens in the cause and cure of cancer and highlight emerging interest in the role of non-oncolytic, non-oncogenic pathogens in cancer progression and treatment.

## Oncogenic Viruses

In 1911, Rockefeller Institute (now Rockefeller University in New York City) pathologist Francis Peyton Rous demonstrated that sarcomas developed in chickens that were administered supernatant of tumor extracts from sarcomas originating in other chickens of the same variety [7]. Tumor extracts were suspended in sterile sand and Ringer’s solution and subjected to multiple rounds of centrifugation, convincing Rous that tumor extracts injected into naïve animals were indeed cell-free [7]. Rous proposed that the transmissible, cancer-causing agent was a “minute parasitic organism” or a “chemical stimulant elaborated by the neoplastic cells” [7]. However, contemporaries dismissed these ideas and insisted that the tumor extracts were not completely cell-free and/or that the masses Rous identified were infectious granulomata rather than tumors [3]. The importance of the Rous sarcoma virus (RSV), cancer-causing agent derived from the cell-free supernatant of chicken tumor extracts, was overlooked until 1958, when Howard Temin and Harry Rubin demonstrated that RSV could convert a chicken fibroblast into a cell with an embryonic cell phenotype [3, 8]. This key experiment brought forth a revival of the study of RSV, and Rous was awarded the Nobel Prize in Physiology and Medicine in 1966 [3]. In 1970, Howard Temin, Satoshi Mizutani, and David Baltimore discovered that the RSV virion harbored an RNA-dependent DNA polymerase, in addition to its single-stranded RNA genome [9, 10]. Viruses possessing the capability of converting RNA to DNA, now called retroviruses, were later shown to use an enzyme called integrase to insert viral DNA into the host genome [11]. RSV was found to contain in its genome an avian-derived oncogene [12], *src*, and the integration of *src* into a host cell genome was demonstrated to be the mechanistic driver of oncogenesis by RSV [13]. Apart from such integration of a host cell-derived oncogene into the host genome, it has been widely acknowledged that another mechanism of retroviral-induced carcinogenesis is integration of the viral genome in regions of proto-oncogenes or tumor suppressor genes. Disruptions in such genes may yield hyperactivity of a proto-oncogene or loss of function of tumor suppressor gene products [14]. RSV is an example of an oncogenic virus, that is, a virus causally linked to the development of cancer. The cancer-causing genetic alterations induced by RSV infection constitute a direct mechanism for oncogenesis. While RSV serves as a classic example by which viruses can promote tumor development by introduction of an oncogene into the host genome, there are countless other oncogenic infections that induce cancer development by indirect means.

Oncogenic viruses are not just limited to retroviruses and vary widely in the mechanisms by which they induce tumorigenesis. Another significant, albeit indirect, mechanism by which viruses can cause cancer is via damage induced by the inflammation and perpetual cell turnover associated with chronic infection [15]. Hepatitis B virus (HBV) and hepatitis C virus (HCV), both implicated in the development of hepatocellular carcinoma (HCC), are two viruses that are oncogenic, mainly as a consequence of chronic inflammation in the liver [16]. Typically, HCC arises in patients infected with hepatitis for two decades or longer, who usually present with cirrhosis or severe fibrosis prior to HCC diagnosis [17]. Research has shown that cytokines that are present at constitutively high levels as a product of chronic inflammation, such as TNF-α and IL-6, may promote tumor cell proliferation [18]. IL-6 operates as a growth factor for cancer via its downstream activation of the transcription factor STAT3 [18]. Interestingly, IL-6 has been implicated as a predictor of whether a patient with chronic viral hepatitis infection progresses to HCC, with patients exhibiting higher serum levels of IL-6 more frequently developing HCC than those harboring lower levels [19]. Although the indirect mechanism of chronic inflammation as an agent of tumorigenesis is believed to be a main driver of HCC development in individuals with chronic HBV or HCV infection, there is some evidence that these viruses mediate oncogenesis directly. For example, the HBx protein encoded by HBV is known to upregulate the MAP/ERK pathway and lead to genomic instability [20]. Nevertheless, the inflammation brought forth by chronic infection is a clinically significant mechanism of oncogenic virus-mediated tumorigenesis. In fact, inflammation as a mediator of cancer development is not limited to oncogenic viral infection. Individuals with Crohn’s disease have an elevated risk of developing colorectal cancer [21], and those infected with the bacterium *Helicobacter pylori* are at higher risk than the general population for developing stomach cancer [22].

While commonly referenced as classic mechanisms by which viruses can cause cancer, retroviral integration into the host genome and inflammation and cell turnover caused by chronic viral infection are only two of the several mechanisms by which viral infection can impact tumor development. Epstein-Barr virus (EBV) has been linked to nasopharyngeal carcinoma and several subtypes of lymphoma, including Burkitt’s lymphoma and Hodgkin’s lymphoma [23]. EBV is a double-stranded DNA gammaherpes virus that infects B cells and later establishes latency [23]. EBV-infected B lymphocytes acquire an activated phenotype, engaging survival-promoting B cell signaling, and consequently, leading to tumorigenesis [23]. Merkel cell polyomavirus, the virus responsible for most Merkel cell carcinomas observed in humans, has been shown to induce transformation in rodents, in vivo, under certain conditions [24]. One mechanism by which polyomaviruses promote the development of cancer is by inducing the host cell to transition into the S phase of the cell cycle, promoting host cell division, and consequently, viral replication [25]. Another double-stranded DNA virus, human papillomavirus (HPV) [26], which can cause cervical cancer and head and neck cancer, mediates tumorigenesis by other mechanisms. Specifically, degradation of the pro-apoptotic protein Bak is mediated by HPV protein E6, conditioning pre-cancerous cells for survival [27]. Furthermore, E6 is believed to disrupt normal functioning of tumor suppressors, such as p53 [27].

Oncogenic infections can also increase the incidence of cancer in the context of immunosuppression. The best-known example of this is observed in individuals infected by human immunodeficiency virus (HIV). Patients with HIV have elevated incidence of several cancers referred to as acquired immunodeficiency syndrome (AIDS)-defining cancers (ADCs) subsequent to HIV infection including Kaposi’s sarcoma, primary central nervous system lymphoma (PCNSL), cervical cancer, and non-Hodgkin’s lymphoma [28]. Additionally, other types of cancers referred to as non-AIDS-defining cancers (NADCs) such as lung, liver, anal, and melanoma are increased in HIV-infected individuals and are major contributors to morbidity and mortality in this patient population. In the context of HIV infection-induced global immunosuppression, oncogenic viruses such as EBV can establish chronic infection, leading to stimulation of B cells that can drive the development of lymphoma, as previously discussed [28]. Approximately 80–100% of Hodgkin’s lymphomas and PCNSLs in AIDS patients can be attributed to EBV infection [28]. Hepatitis B and hepatitis C infections, as well as human papillomavirus infections, have been reported at high frequency in AIDS patients, and observed in conjunction with hepatocellular and cervical cancers, respectively [28]. These data illustrate that loss of immunological control of viral infection is strongly linked to the development of cancer, indicating the major role that pathogens play in promoting tumorigenesis. Furthermore, it should be noted that the emergence of cancer in AIDS individuals can be partially attributable to the inability of an immunocompromised individual to mount functional anti-tumor immune responses against tumors in their nascent, subclinical stages [29]. The ability of the immune system to detect abnormal-self cells is dependent upon several physiological factors, including the presence or absence of viral infection, as is discussed in the sections below. Further, in specific contexts, non-oncolytic, non-oncogenic viral infections can be exploited to augment anti-tumor immune responses.

## Oncolytic Viruses

Mechanistically, it is thought that the therapeutic potential of oncolytic virus infection relies on two main actions of such viruses, first being preferential lysis of tumor cells and the second being the resultant priming of a systemic anti-tumor immune response subsequent to cell lysis-mediate release of tumor antigens in the context of inflammation [2]. Defective interferon and toll-like receptor signaling in tumor cells allows for successful viral replication, while non-cancerous cells equipped with functional interferon signaling and other viral recognition pathways effectively thwart viral replication, thereby mainly limiting oncolytic viral infection to tumor cells [2, 30]. Failure to clear virus from tumor cells can result in activation of apoptosis, necrosis, or pyroptosis, yielding lysis of such tumor cells [2]. Upon lysis, tumor neoantigens, pattern-associated molecular patterns (PAMPs; such as viral proteins and genomic material), and damage-associated molecular patterns (DAMPs) such as ATP, calreticulin, and uric acid, are released from the cell [2]. Released antigens are engulfed and presented by antigen presenting cells, leading to the activation of IL-2-secreting CD4^+^ T lymphocytes [2]. Engagement of IL-2 by the IL-2 receptor on cytotoxic T (CD8^+^) lymphocytes yields activation of CTLs reactive to tumor antigens [2]. Cytokines such as TNF-α, IFN-γ, and IL-12 released from lysed tumor cells can engage cytokine receptors on natural killer (NK) and CD8^+^ T cells, promoting destruction of tumor cells that downregulate major histocompatibility complex (MHC) antigen-presentation molecules and tumor cells expressing neoantigens, respectively [2]. In summation, oncolytic viruses can promote tumor cell death by inducing lysis of infected cells, exposing tumor-associated antigens, neoantigens, and danger signals that can subsequently initiate an anti-tumor immune response.

In the clinic, oncolytic viruses have demonstrated efficacy in curtailing tumor growth. In October 2015, the United States Food and Drug Administration (FDA) approved Imlygic (Amgen, Inc.), a modified herpes simplex-1 (HSV-1) oncolytic virus therapy [31]. Imlygic, also called talimogene laherparepvec (T-VEC), harbors a deletion in the ICP34.5 neurovirulence gene and the ICP47 gene, which obstructs antigen presentation [32]. Further, an insertion of the granulocyte macrophage-colony stimulating factor (GM-CSF) gene yields infiltration of macrophages and dendritic cells into the infected tumor, thereby strengthening the anti-tumor immune response [32]. The results of a 436-patient clinical trial comparing intratumoral administration of T-VEC to subcutaneously delivered GM-CSF in patients with stage IIIb to IV melanoma, published in 2015, indicated that 16.3% of T-VEC-treated patients had a durable response to therapy, compared to the 2.1% durable response rate observed for GM-CSF treatment [33]. Further, median survival was increased in the T-VEC-treated arm. Together, these data demonstrate that T-VEC can be employed as a treatment for melanoma, without an excess of detrimental side effects [33].

Oncolytic virus therapies utilizing other classes of virus and various genetic modifications are currently under investigation and in clinical trial. Coxsackievirus, vaccinia virus, adenovirus, reovirus, Newcastle disease virus, measles virus, and others have been candidates for oncolytic virus therapy of cancer [34]. Cytokines and molecular mediators of the immune system that can augment the immune response initiated by oncolytic viral infection include IL-2, IL-12, IL-15, IFN-α, and 4-1BB. Genes encoding these cytokines have been explored as potential candidates for insertion into oncolytic viral genomes. In China, a modified adenovirus, H101, was approved in 2006 for head and neck squamous cell carcinoma [35]. Coxsackievirus A21 (CVA21) has been tested as an oncolytic viral therapy for melanoma with one clinical trial already demonstrating that 19.3% of patients exhibit a durable response, and 75.4% of patients survive 1 year after beginning treatment [36]. Additionally, an attenuated poliovirus (PVSRIPO) harboring the internal ribosome entry site of human rhinovirus type 2 (HRV2) is currently under investigation for the treatment of glioblastoma [37]. Glioblastoma cells largely express CD155 (the receptor for poliovirus), rendering them a good target for PVSRIPO oncolytic virus therapy [38]. In conjunction with use of other immunotherapies, such as PD-1 and CTLA-4 blockade, oncolytic viruses have the potential to become even more effective treatments for cancer. For a summary of select clinical trials of oncolytic viruses with clinical and immune outcomes data, please see the following reviews [39, 40]. Please visit clinicaltrials.gov for a continuously updated comprehensive list.

## Non-Oncolytic, Non-Oncogenic Viruses

Oncogenic and oncolytic viruses employ defined mechanisms to cause or curtail cancer, respectively. The impact of infection by non-oncogenic, non-oncolytic viruses on tumor development and progression is less understood. In 1990, it was found that C57BL/6 mice intravenously challenged with lymphocytic choriomeningitis virus (LCMV) exhibited faster growth of intradermal B16 melanoma than uninfected controls [41]. Contrarily, it has been reported that influenza-experienced mice exhibit slower growth of intradermally injected 3LL, a Lewis lung carcinoma cell line, than influenza-naïve counterparts, suggesting that infection confers protection from cancer [42]. Conflicting data regarding the link between non-oncogenic, non-oncolytic viral infection and cancer has been identified among epidemiological studies. Reports have shown that certain infections may increase rates of cancer-specific death for particular subtypes of cancer, while other viral infections may yield accelerated cancer-related death in other clinical contexts [43, 44]. In 2016, we published that mice challenged prior to clinical tumor emergence with intranasally administered PR8/H1N1/influenza A exhibit accelerated melanoma growth and accelerated cancer-specific death than uninfected melanoma-bearing mice [45]. Cytotoxic T lymphocytes reactive against a tumor antigen were found at a high frequency at the tumor site in uninfected animals. In mice concomitantly challenged with influenza infection, such tumor-reactive cells were found at a high frequency at the site of infection, the lungs [45]. These data suggest that tumor-specific cytotoxic T lymphocytes, which are indispensable for control of cancer, may migrate to the lung, the site of influenza infection, in mice co-challenged with influenza and melanoma. Mechanistically, non-oncogenic, non-oncolytic viral infection may impact the development of tumors in distant anatomical locations by serving as an immunological distraction.

## Conclusions and Future Directions

Further research into the mechanisms that govern tumor development and cancer cure in the context of oncogenic and oncolytic viruses, respectively, is needed. Particularly, it is important to deconstruct the mechanisms underlying the actions of such pathogens to understand the indirect effects that they exert on the immune responses to cancer. Just as the role of infections is two-fold leading to cancer promotion and regression, these indirect effects are likely to be two-fold leading to distraction of the immune response away from the tumor and in reversing the immunosuppressive nature of the tumor microenvironment. Towards developing cures for cancer, it is important that the future of research in this field be focused on this latter effect, specifically on harnessing anti-pathogen immune responses as a means to treat cancer.
